# Effects of Metabolic Energy on Synaptic Transmission and Dendritic Integration in Pyramidal Neurons

**DOI:** 10.3389/fncom.2018.00079

**Published:** 2018-09-26

**Authors:** Ye Yuan, Hong Huo, Tao Fang

**Affiliations:** ^1^Department of Automation, Shanghai Jiao Tong University, Shanghai, China; ^2^Key Laboratory of System Control and Information Processing, Ministry of Education, Shanghai, China

**Keywords:** synaptic transmission, dendritic integration, metabolic energy, release probability, connection number, biologically plausible models, synaptic vesicle cycle

## Abstract

As a sophisticated computing unit, the pyramidal neuron requires sufficient metabolic energy to fuel its powerful computational capabilities. However, the majority of previous works focus on nonlinear integration and energy consumption in individual pyramidal neurons but seldom on the effects of metabolic energy on synaptic transmission and dendritic integration. Here, we developed biologically plausible models to simulate the synaptic transmission and dendritic integration of pyramidal neurons, exploring the relations between synaptic transmission and metabolic energy and between dendritic integration and metabolic energy. We find that synaptic energy not only drives synaptic vesicle cycle, but also participates in the regulation of this cycle. Release probability of synapses adapts to synaptic energy levels by regulating the speed of synaptic vesicle cycle. Besides, we also find that to match neural energy levels, only a part of the synapses receive presynaptic signals during a given period so that neurons have a low action potential frequency. That is, the number of simultaneously active synapses over a period of time should be adapted to neural energy levels.

## Introduction

The widely distributed pyramidal neurons in the cerebral cortex of mammals are highly critical to cognition and memory (Spruston, 2008). Synaptic transmission and dendritic integration are two important steps in information processing in a single pyramidal neuron. During synaptic transmission, action potentials from the axon initial segment (AIS) induce synaptic vesicles to release neurotransmitter into synaptic clefts. Then, the released neurotransmitter binds to receptors on the postsynaptic membrane, causing a change in the postsynaptic membrane potential (Engelman and Macdermott, 2004). Subsequently, the intricate dendrites of postsynaptic neurons efficiently collect thousands of presynaptic signals and integrate them through both linear and nonlinear mechanisms (Silver, 2010; Grienberger et al., 2015; Stuart and Spruston, 2015). Existing studies have shown that synaptic transmission and dendritic integration are metabolically expensive, while the metabolic energy of neurons is limited (Attwell and Laughlin, 2001; Lennie, 2003; Howarth et al., 2012; Yi et al., 2017). Therefore, neurons in the central nervous system (CNS) should coordinate the relation between these neural activities and metabolic energy levels during synaptic transmission and dendritic integration (Hasenstaub et al., 2010). Unfortunately, few previous studies focus on this relation. During synaptic transmission, the higher release probability of synapses means that more information can be transmitted to postsynaptic neurons, but many experiments show that the majority of synapses maintain a relatively low release probability to obtain optimal energy efficiency (Goldman, 2004; Volgushev et al., 2004; Harris et al., 2012). A large number of synaptic connections can ensure that neurons produce high-frequency action potential signals, but some studies indicate that regardless of how the dendrites of pyramidal neurons bifurcate, homeostatic plasticity can maintain the frequency of action potentials within a fixed range (Turrigiano, 1999; Spruston, 2008). Therefore, we believe that stable relations may exist between the release probability of synapses and metabolic energy levels and between the number of simultaneously active synapses over a period of time and metabolic energy levels. In this paper, we first propose a novel synaptic transmission model to explore the relation between synaptic vesicle cycle and metabolic energy levels, and then establish a multi-compartment model to study the relation between the number of simultaneously active synapses over a period of time and metabolic energy levels.

## Methods

### Model of the synaptic vesicle cycle

**Figure 1:**
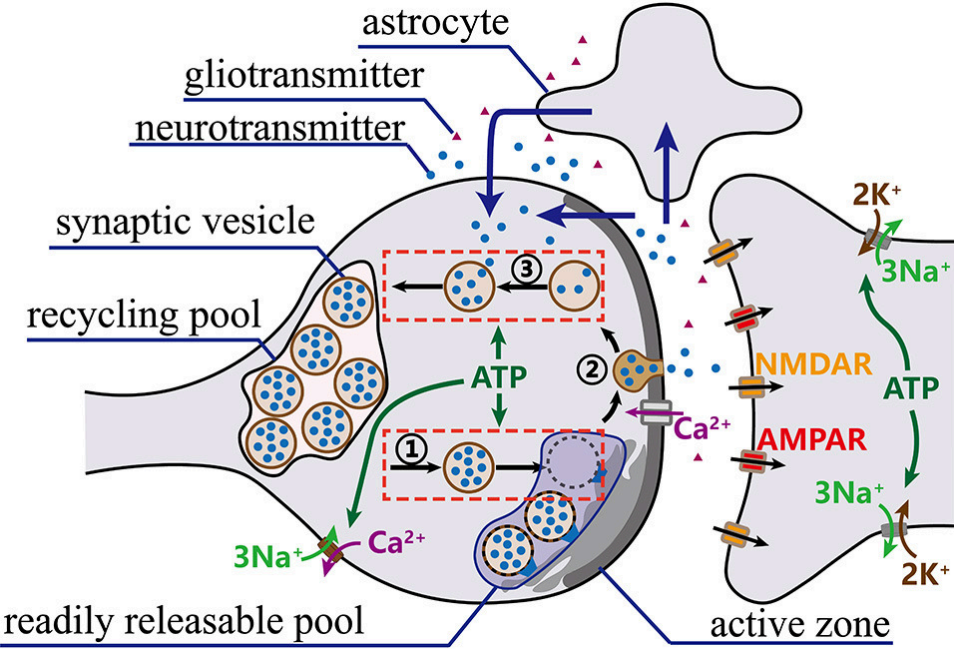
The synaptic vesicle cycle. Prepared synaptic vesicles are marked with a dark color, while unprepared ones are marked with a light color. Once action potentials arrive at synaptic terminals, vesicles in RRP successively move to presynaptic membrane to fuse with release sites and release neurotransmitters into the cleft. Meanwhile, vesicles from the recycling pool quickly move to refill the RRP. Then, the released neurotransmitters bind to receptors on the postsynaptic membrane to complete signal transmission. Finally, most of the released neurotransmitters are transported back to the presynaptic terminals directly or via astrocytes (blue arrows) and taken up into synaptic vesicles for a new round of exocytosis.

Synaptic vesicles containing neurotransmitters are mainly distributed in the reserve pool, the recycling vesicle pool and the readily releasable pool (RRP) (Rizzoli and Betz, 2005). Recent evidence indicates that vesicles in the recycling and reserve pools are intermixed to a considerable degree (Sudhof, 2004; Rizzoli and Betz, 2005; Südhof, 2013). Therefore, our synaptic transmission model only considers the recycling pool and the readily releasable pool, as shown in Figure 1. Synaptic transmission involves sophisticated synaptic vesicle cycle, which can be roughly divided into three stages (Sudhof, 2004; Schweizer and Ryan, 2006): (1) synaptic vesicles moving from the recycling pool to the RRP and preparing for exocytosis; (2) synaptic vesicles fusing with the presynaptic membrane and releasing neurotransmitters; and (3) synaptic vesicles recycling neurotransmitters and returning to the recycling pool. Here, we establish a novel synaptic transmission model to describe all the stages of synaptic transmission.

First, only prepared synaptic vesicles in the RRP are generally believed to be able to release neurotransmitters (Sudhof, 2004; Schweizer and Ryan, 2006). Once prepared synaptic vesicles fuse with the presynaptic membrane, the same number of synaptic vesicles from the recycling pool quickly move to refill the RRP (Rizzoli and Betz, 2005; Alabi and Tsien, 2012). The number of synaptic vesicles in the RRP accounts for approximately 5~10% of the total number of synaptic vesicles in a presynaptic terminal (Alabi and Tsien, 2012). Assuming that the RRP contains at most *N*_*RRP*_ synaptic vesicles, the preparation of synaptic vesicles for the release of neurotransmitters can be described as follows:

(1) $$\begin{array}{l} \tau_{s}\frac{ds_{i}}{dt}+s_{i}=s_{m},\left(s_{i}{|}_{t=0}=0,i=1,2,\cdots \;,N_{RRP}\right) \end{array}$$

where *s*_*i*_ represents the state of the *i*-th synaptic vesicle at time *t*, and *s*_*i*_ = 0 and *s*_*i*_ = *s*_*m*_ represent its initial state and its state ready for release, respectively. For any RRP synaptic vesicle, *s*_*i*_ increases from 0 to *s*_*m*_ during the preparation for exocytosis, where *s*_*m*_ is a dimensionless constant whose value can be any real number greater than 0. The time constant τ_*s*_ determines the change rate of the state of RRP synaptic vesicles. When the time constant τ_*s*_ is larger, the state value changes more slowly; otherwise, the state value changes more quickly. In other words, the rate of the vesicles preparing for exocytosis in our model depends uniquely on the value of the time constant τ_*s*_.

Second, after preparation is complete, synaptic vesicles fuse with the free release sites and release neurotransmitters (Sudhof, 2004; Schweizer and Ryan, 2006). In this stage, the number of release sites is limited and ATP concentration has little influence on this phase (Heidelberger et al., 2002; Neher, 2010). For simplicity, the maximum usage per release site and the maximum usage of a synapse both can be assumed to be fixed. The maximum usage per release site refers to the maximum number of synaptic vesicles which can release neurotransmitter through a release site per second, and the maximum usage of a synapse refers to the maximum number of synaptic vesicles which can release neurotransmitter through all release sites of a synapse per second. Furthermore, action potentials induce a large amount of Ca^2+^ to pour into neuron terminals. The synaptic vesicles can release neurotransmitters only when the total Ca^2+^ concentration in synaptic terminals reaches a certain level (Sheng et al., 1998; Schweizer and Ryan, 2006). After entering synaptic terminals, the majority of Ca^2+^ quickly binds to proteins at plasma membranes, while the remaining free Ca^2+^ is rapidly excluded by 3Na^+^/Ca^2+^ ion pumps and Ca^2+^-ATPase (Helmchen et al., 1997). The rising phase of the free Ca^2+^ concentration is much shorter than the counterpart descending stage. In the proposed model, the free Ca^2+^ concentration is calculated as follows (Helmchen et al., 1997; Lu et al., 2016),

(2) $${\left[{\text{Ca}}^{2+}\right]}_{\text{i}}=\{\begin{array}{l} {\left[{\text{Ca}}^{2+}\right]}_{\text{rest}}+\Delta {\left[{\text{Ca}}^{2+}\right]}_{\text{AP}}\cdot {\text{e}}^{-\text{t}/\tau \text{Ca}},\left(\text{t}\geq {\text{t}}_{\text{AP}}\right)\\ {\left[{\text{Ca}}^{2+}\right]}_{\text{rest}}\;,\left(\text{t}<{\text{t}}_{\text{AP}}\right) \end{array}$$

where [Ca^2+^]_i_ is the free Ca^2+^ concentration, and [Ca^2+^]_rest_ is the free Ca^2+^ concentration at rest. Δ[Ca^2+^]_AP_ is the change in the free Ca^2+^ concentration in response to a single action potential, and t_AP_ is the time at which an action potential arrives at the synaptic terminal. τ_Ca_ is the decay time constant of the free Ca^2+^ concentration. Every action potential can cause the free Ca^2+^ concentration to increase quickly and then to decline exponentially. It takes approximately 100 ms for the free Ca^2+^ concentration to recover to its original level before the action potential arrives (Helmchen et al., 1997). The change in the total Ca^2+^ concentration is calculated as follows (Helmchen et al., 1997; Lu et al., 2016),

(3) $$\Delta {\left[{\text{Ca}}^{2+}\right]}_{\text{total}}=\Delta {\left[{\text{Ca}}^{2+}\right]}_{\text{AP}}\cdot \left(1+{\text{K}}_{\text{s}}+{\text{K}}_{\text{B}}^{\prime }\right)$$

where Δ[Ca^2+^]_total_ is the total Ca^2+^ concentration, Ks is the endogenous Ca^2+^ binding ratio, and ${\text{K}}_{\text{B}}^{\prime }$ is the incremental Ca^2+^ binding ratio for the exogenous Ca^2+^ buffer. Δ[Ca^2+^]_AP_ is the same as in Equation (2). Previous studies have shown that the neurotransmitter release is initiated by influx of Ca^2+^ within 200 μs of the action potential arriving at the synaptic terminal, and the exocytosis of vesicles requires high Ca^2+^ concentration, with a threshold of 20~50 μmol/L (Sheng et al., 1998; Fernándezalfonso and Ryan, 2004). For simplicity, the total Ca^2+^ concentration in the model is regarded as an indicator that determines whether neurotransmitters can be released or not. When the total Ca^2+^ concentration exceeds the threshold, synaptic vesicles start cycling and neurotransmitters are released. Otherwise, synaptic vesicles stop cycling and neurotransmitters cannot be released. It is worth noting that in the proposed model, the total Ca^2+^ concentration below the threshold does not mean that synaptic vesicles are inactive. Although synaptic vesicles cannot release neurotransmitters when the total Ca^2+^ concentration is below the threshold, they are still preparing for neurotransmitter release and slowly fusing with release sites. These prepared synaptic vesicles can immediately release neurotransmitters as soon as the total Ca^2+^ concentration exceeds the threshold again. Besides, when an action potential arrives at the synaptic terminal, the total Ca^2+^ concentration will remain above the threshold for a period of time during which the neurotransmitter can be released.

Third, most released neurotransmitters are transferred back to presynaptic terminals directly or via astrocytes and then are taken back by empty synaptic vesicles that are returning to the recycling pool (Schweizer and Ryan, 2006). ATP hydrolysis is also required in this process (Chapman, 2008). Furthermore, the existing research results indicate that the return rate of synaptic vesicles to the recycling pool decreases as the number of synaptic vesicles approaches the maximum capacity of the recycling pool (Heidelberger et al., 2002). Therefore, the return process of synaptic vesicles can be approximately described as follows

(4) $$\begin{array}{l} v_{r}\cdot \tau_{r}=h_{m}-h\left(t\right),\text{if }v_{r}>v_{rm},v_{r}\leftarrow v_{rm} \end{array}$$

where *v*_*r*_ and *v*_*rm*_ represent the actual return rate and the maximum return rate of synaptic vesicles to the recycling pool, respectively, and *h*_*m*_ and *h(t)* represent the maximum number and the actual number of synaptic vesicles in the recycling pool, respectively. The return rate of vesicles to the recycling pool is closely related to the actual number of vesicles in the pool. A greater difference between the actual and maximum number of vesicles indicates a greater return rate. The time constant τ_*r*_ determines the change rate of the return rate *v*_*r*_. When the time constant τ_*r*_ is larger, the return rate changes more slowly; otherwise, the return rate changes more quickly. It is worth noting that the return rate cannot increase without limit. For example, in the calyx of Held nerve terminals, the maximum endocytosis rate of empty synaptic vesicles is 1.8 per second at 37°C (Neher, 2010). Therefore, we assume that the return rate of synaptic vesicles to pools must be less than the maximum rate *v*_*rm*_.

Different numbers of synaptic vesicles are stored in different types of pools (Rizzoli and Betz, 2005). However, for a given vesicle pool, the capacity of the pool remains at a constant level, and the actual number of vesicles in the pool is dependent on time. The depletion kinetics of the recycling pool can be described as follows

(5) $$\begin{array}{l} h\left(t\right)=h_{m}+\int_{0}^{t}\left(v_{r}\left(\tau \right)-v_{s}\left(\tau \right)\right)d\tau \end{array}$$

where *v*_*r*_, *h*_*m*_ and *h(t)* are the same as in Equation (4), and *v*_*s*_ represents the rate of synaptic vesicles leaving the recycling pool. The integral of the difference between *v*_*r*_ and *v*_*s*_ in a period represents the change in the number of vesicles in the recycling pool during the period.

To describe a complete synaptic vesicle cycle, we use the above four formulas to construct a novel synaptic transmission model in which the input is the number of action potentials and the output is the number of synaptic vesicles. The biological experiments show that the energy expended per vesicle of neurotransmitter released is 1.64 × 10^5^ ATP molecules (Attwell and Laughlin, 2001; Yu et al., 2017). Therefore, the energy consumption over a period of time can be described as follows

(6) $$\begin{array}{l} E_{trans}^{a}=1.64\times 10^{5}\cdot N_{vesicle} \end{array}$$

where $E_{trans}^{a}$ and *N*_*vesicle*_ represent the actual energy consumption of synapses and the number of released vesicles over a period of time, respectively. Note that it is difficult to estimate the number of calcium ions and recycled neurotransmitters when action potentials continuously arrive at synaptic terminals. Therefore, for simplicity, the calculation for the energy consumption of calcium ions and neurotransmitter recycling is ignored, and the total energy expended per vesicle of neurotransmitter released is treated as a constant. We also pay attention to the release probability of synapses. For a synapse with a single release site, the release probability refers to the probability that a single action potential arrival at synapse will result in neurotransmitter release (Stevens, 2003; Harris et al., 2012). In our model, the average number of released vesicles induced by a single action potential is computed, and then the release probability of synapses is computed as follows

(7) $$\begin{array}{l} rp=\frac{Q}{N} \end{array}$$

where *rp* represents the release probability of synapses. *N* represents the number of release sites contained by a synapse. *Q* represents the average number of released vesicles induced by a single action potential over the period of time. Note that under physiological conditions, a release site can only release a single vesicle following arrival of an action potential (Stevens, 2003; Harris et al., 2012). That is, the release probability is certainly less than 1.0. However, in the synaptic transmission model, it is possible that the release probability is greater than 1.0. In this case, the release probability is thought to be abnormal. Based on previous studies (Rizzoli and Betz, 2005; Neher, 2010), a single synapse in the model is assumed to contain 200 synaptic vesicles and 5 release sites in total. The optimal range of the release probability of synapses in the model is set to 0.25~0.5 according to the existing experimental findings (Harris et al., 2012). In addition, because the maximum usage per release site is ~3.5 vesicles per second (Neher, 2010), the maximum usage of a synapse is assumed to be 20 vesicles per second. Synaptic transmission model is implemented in MATLAB and its code is available upon request.

### Regulation on synaptic vesicle cycle by metabolic energy in synaptic transmission model

To investigate the effects of metabolic energy on synaptic transmission, a necessary and fundamental step is to develop a framework in which the cycling of synaptic vesicles is closely associated with synaptic energy level. As we know, in the process of synaptic vesicle cycle, synaptic vesicles preparing for exocytosis and returning to the recycling pool both need ATP hydrolysis to provide metabolic energy, which means that these two processes are affected by metabolic energy (Heidelberger et al., 2002). Besides, existing studies also show that the synaptic function of neurons must be matched with their metabolic energy level at synapses, and metabolic energy could exert constraints over the synaptic function of neurons at different levels (Göbel et al., 2010; Rangaraju et al., 2014). Therefore, a constraint is introduced into the model to achieve the regulation on synaptic vesicle cycle by metabolic energy. In the model, although synaptic vesicles preparing for exocytosis and returning to the recycling pool both present large ATP demands, the regulation of these two processes exerts different effects on the energy consumption at synapses. Before the synaptic vesicles in the recycling pool are depleted, regulating the process of synaptic vesicles preparing for exocytosis could change synaptic energy consumption, while regulating the process of synaptic vesicles returning to the recycling pool would exert little effect on synaptic energy consumption. In fact, under physiological conditions, the synaptic vesicles in the recycling pool are rarely depleted (Fernándezalfonso and Ryan, 2004). Therefore, for simplicity, only the process of synaptic vesicles preparing for exocytosis is regulated by metabolic energy in our model.

These raise a question about how to regulate the process of synaptic vesicles preparing for exocytosis according to synaptic energy levels. Here, from the optimization principle, we derive an essential equation which describes the basic relation between synaptic vesicle cycle and synaptic energy level. In our model, the time constant τ_*s*_ defined in Equation (1) uniquely determines the rate of synaptic vesicle preparing for exocytosis, which also indicates that the speed of synaptic vesicle cycle is closely related to the time constant τ_*s*_. Therefore, we can regard the energy consumption of synaptic vesicle cycle over a period of time as a function of the time constant τ_*s*_. The energy optimization of synaptic vesicle model can be abstracted as a constraint

(8) $$\begin{array}{l} \min f\left(\tau_{s}\right)\\ st.f\left(\tau_{s}\right)=|E_{trans}^{a}-E_{trans}^{d}|,\\ E_{trans}^{a}>0,E_{trans}^{d}>0. \end{array}$$

where $E_{trans}^{a}$ and $E_{trans}^{d}$ represent the actual energy consumption and desired energy consumption at synapses over a period of time, respectively. The desired energy consumption is set manually to reflect the level of metabolic energy at synapses. According to optimization principles, we can use gradient descent algorithm

(9) $$\begin{array}{l} \tau_{s}^{\left(k+1\right)}=\tau_{s}^{\left(k\right)}-\lambda_{k}\cdot \nabla f\left(\tau_{s}^{\left(k\right)}\right) \end{array}$$

to iteratively update τ_*s*_ to minimize *f*(τ_*s*_), where λ_*k*_ is the iterative step size. Unfortunately, from an engineering point of view, synapse is a complex nonlinear time-varying system, and we cannot obtain accurate analytical formula of *f*(τ_*s*_). We note that a shortage of synaptic energy can result in the deceleration of the cycle, which in turn promotes the recovery of synaptic energy (Heidelberger et al., 2002). Obviously, there is a negative relationship between the cycle and synaptic energy. Therefore, we can infer that τ_*s*_ is inversely proportional to $E_{trans}^{a}$, and $\left(E_{trans}^{a}-E_{trans}^{d}\right)$ is proportional to $\nabla f\left(\tau_{s}^{\left(k\right)}\right)$. To avoid the need to obtain accurate analytical formula of *f*(τ_*s*_), $\left(E_{trans}^{a}-E_{trans}^{d}\right)$ is used to replace $\nabla f\left(\tau_{s}^{\left(k\right)}\right)$. Considering the large difference in magnitude between $\left(E_{trans}^{a}-E_{trans}^{d}\right)$ and $\nabla f\left(\tau_{s}^{\left(k\right)}\right)$, we modify $\left(E_{trans}^{a}-E_{trans}^{d}\right)$ and finally get

(10) $$\begin{array}{l} \tau_{s}^{\left(k+1\right)}=\tau_{s}^{\left(k\right)}+\lambda_{k}\cdot \left(\frac{2}{1+e^{-\left(E_{trans}^{a}-E_{trans}^{d}\right)}}-1\right) \end{array}$$

Obviously, a value of $\left(E_{trans}^{a}-E_{trans}^{d}\right)$ less than 0 indicates a lower ATP concentration at synapses, so the time constant τ_*s*_ should decrease in the next period; otherwise, the time constant τ_*s*_ should increase. According to Equation (10), we can conclude that changes in synaptic vesicle cycle result in fluctuations in synaptic energy level, which in turn affects the cycle of synaptic vesicles. The constraint of synaptic transmission model is implemented in MATLAB, and its code is available upon request.

### Measurement of the information quantity and energy consumption of a spike train

After the synaptic transmission described above, presynaptic signals transmitted through innumerable synapses located at different dendritic branches are propagated into the soma and integrated in linear, supra-linear and sub-linear manners (Magee, 2000; Vetter et al., 2001; Branco and Häusser, 2011; Xu et al., 2012). To study the relation between metabolic energy and dendritic integration, a traditional multi-compartment model is established here (see Supplementary Material). We obtain the action potentials at different frequencies by changing the number of simultaneously active synapses in the model and then calculate the information quantity and energy consumption of a spike train over a period of time according to the action potential frequency. During dendritic integration, pyramidal neurons generate an action potential until the membrane potential reaches the threshold potential; subsequently, ion pumps quickly restore various ion concentrations on both sides of the neuronal membrane to prepare for the next action potential. According to recent studies, action potentials are more energetically efficient than previously thought, and the energy consumption of a single action potential is mainly in the range of (1.1~1.5) × 10^8^ ATP molecules (Harris and Attwell, 2012; Howarth et al., 2012; Yu and Yu, 2017; Yu et al., 2017). Here, for simplicity, the energy consumption of a single action potential is fixed to 1.2 × 10^8^ ATP molecules. Obviously, a higher action potential frequency is indicative of a larger number of ions actively transported per second; consequently, the pyramidal neurons consume energy more rapidly. The information quantity and energy consumption of a spike train can be calculated, respectively, as follows (Harris et al., 2012)

(11) $$\begin{array}{l} I_{info}=\frac{-f\delta \log_{2}\left(f\delta \right)-\left(1-f\delta \right)\cdot \log_{2}\left(1-f\delta \right)}{\delta } \end{array}$$

(12) $$\begin{array}{l} E_{integ}^{a}=1.2\times 10^{8}\cdot f \end{array}$$

where *f* is the frequency of action potential generated by the model. Due to the refractory period, there is a time interval between adjacent action potentials. Here, we use δ to represent the minimum time interval, in which a single action potential is either generated or not. *I*_*info*_ and $E_{integ}^{a}$ represent the information quantity and energy consumption of a spike train per second, respectively.

## Results

### Release probability of synapses adapts to energy level by regulating the speed of the vesicle cycle

Numerous experimental and theoretical studies have demonstrated that a low release probability at hippocampal synapses can maximize the ratio of information transmitted to ATP consumed during synaptic transmission (Levy and Baxter, 2002; Goldman, 2004; Harris et al., 2012). Undoubtedly, the release probability is closely related to the synaptic energy. To investigate the relationship between the release probability and the synaptic energy, a comparative simulation is performed using the synaptic transmission model.

**Figure 2:**
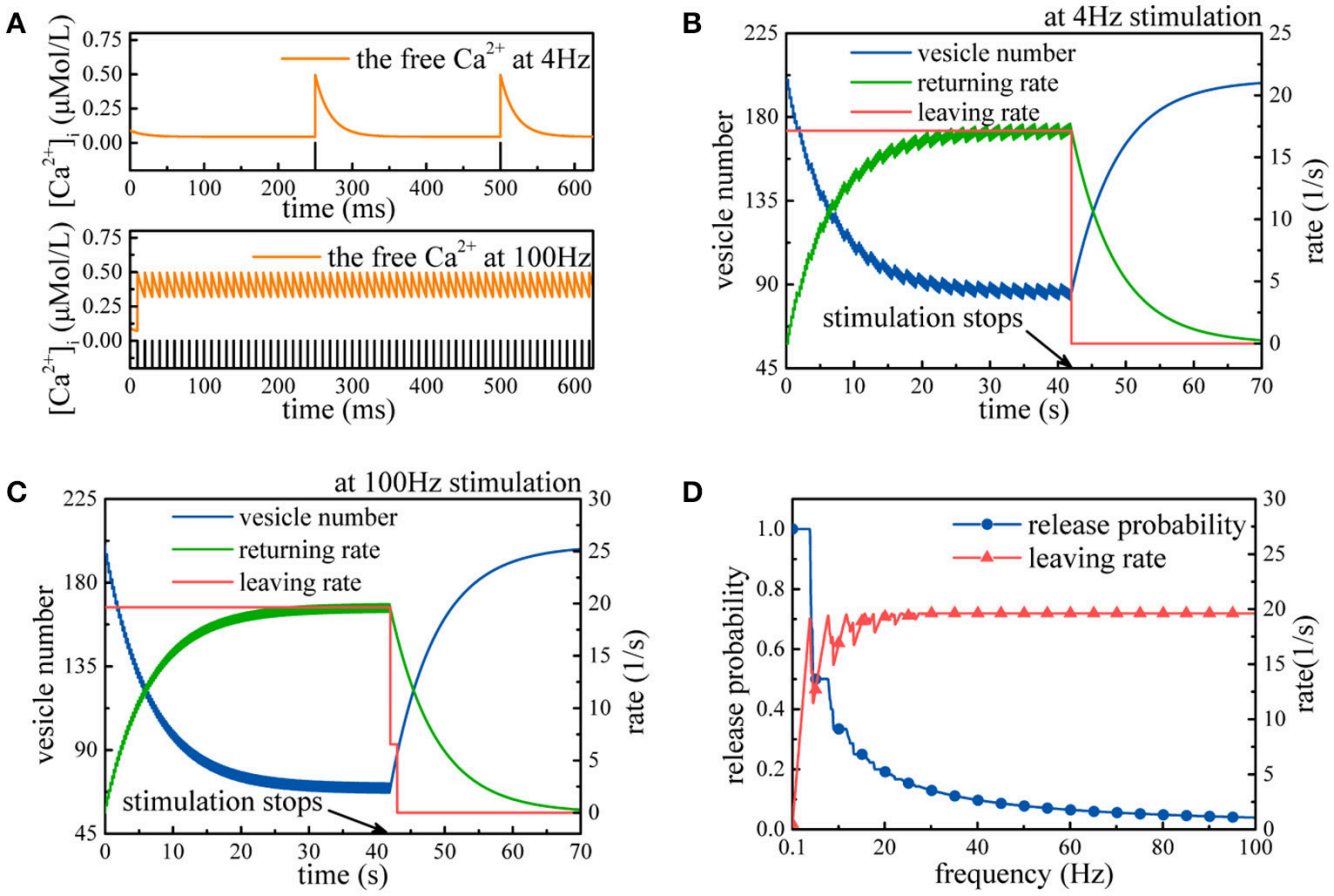
Simulation of the synaptic transmission model without the constraint. **(A)** Fluctuations in the free Ca^2+^ concentration induced by action potentials with 4 and 100 Hz frequencies. **(B,C)** The model receives 4 and 100 Hz stimulation, respectively. Initially, the rate of synaptic vesicles leaving the recycling pool is larger than the rate of synaptic vesicles returning to the recycling pool, so the number of synaptic vesicles in the recycling pool declines. Once *v*_*s*_ equals *v*_*r*_, the number of synaptic vesicles maintains a dynamic balance. After the end of the stimulation, the number of synaptic vesicles in the recycling pool gradually recovers. Notably, synaptic vesicles leave the recycling pool at intervals. **(D)** Action potential sequences with different frequencies are input to the model. Clearly, when the frequency is higher, the release probability is lower, while the leaving rate gradually approaches the maximum usage of a synapse.

First, we simulate synaptic vesicle cycle in the case of not considering the effects of synaptic energy. Two types of action potential sequences (4 and 100 Hz) lead to periodic changes in the free Ca^2+^ concentration (Figure 2A). In both cases, the number of synaptic vesicles in the recycling pool declines until the rate of synaptic vesicles returning to the recycling pool gradually rises to the rate of synaptic vesicles leaving the recycling pool (Figures 2B,C). Once the stimulation stops, the number of synaptic vesicles in the recycling pool gradually returns to the level before the arrival of the action potential. For the 4 Hz stimulation, synaptic vesicles leave the recycling pool at a rate of only 17 synaptic vesicles per second, and the release probability of synapses is about 0.86, while for the 100 Hz stimulation, the leaving rate reaches the maximum usage of a synapse, and the release probability of synapses is about 0.039. This value implies that for the 100 Hz stimulation, all release sites are busy releasing neurotransmitters, and some prepared synaptic vesicles must wait for available release sites. In fact, in the synaptic transmission model, the relative relation between the rate of synaptic vesicles leaving the recycling pool and the usage of a synapse determines the release probability of synapses at different stimulus frequencies. If the rate of synaptic vesicles leaving the recycling pool is greater than the maximum usage of a synapse, the limited number of release sites in the pyramidal neuron leads to the loss of some action potentials.

From Figure 2D, we can find that as the stimulus frequency increases, the rate of synaptic vesicles leaving the recycling pool generally rises with large initial fluctuations and gradually becomes stable until it approaches the maximum usage of a synapse. Meanwhile, the release probability quickly decays as the stimulus frequency increases. Obviously, the release probability heavily depends on the action potential frequency. It is worth noting that the release probability is quite high at low stimulus frequency (Figure 2D). This is inconsistent with existing experimental findings that the release probability of most synapses can always stay within a range of 0.25~0.5 to optimize energy efficiency (Levy and Baxter, 2002; Goldman, 2004; Harris et al., 2012). This conflict between the simulation results and the biological experimental findings is attributed to the absence of the regulation of synaptic energy. Without considering the effects of synaptic energy, the model cannot be optimized according to synaptic energy levels and not necessarily optimal for arbitrary input. Thus, the simulation results is normal for some inputs and abnormal for others, as shown in Figure 2D.

**Figure 3:**
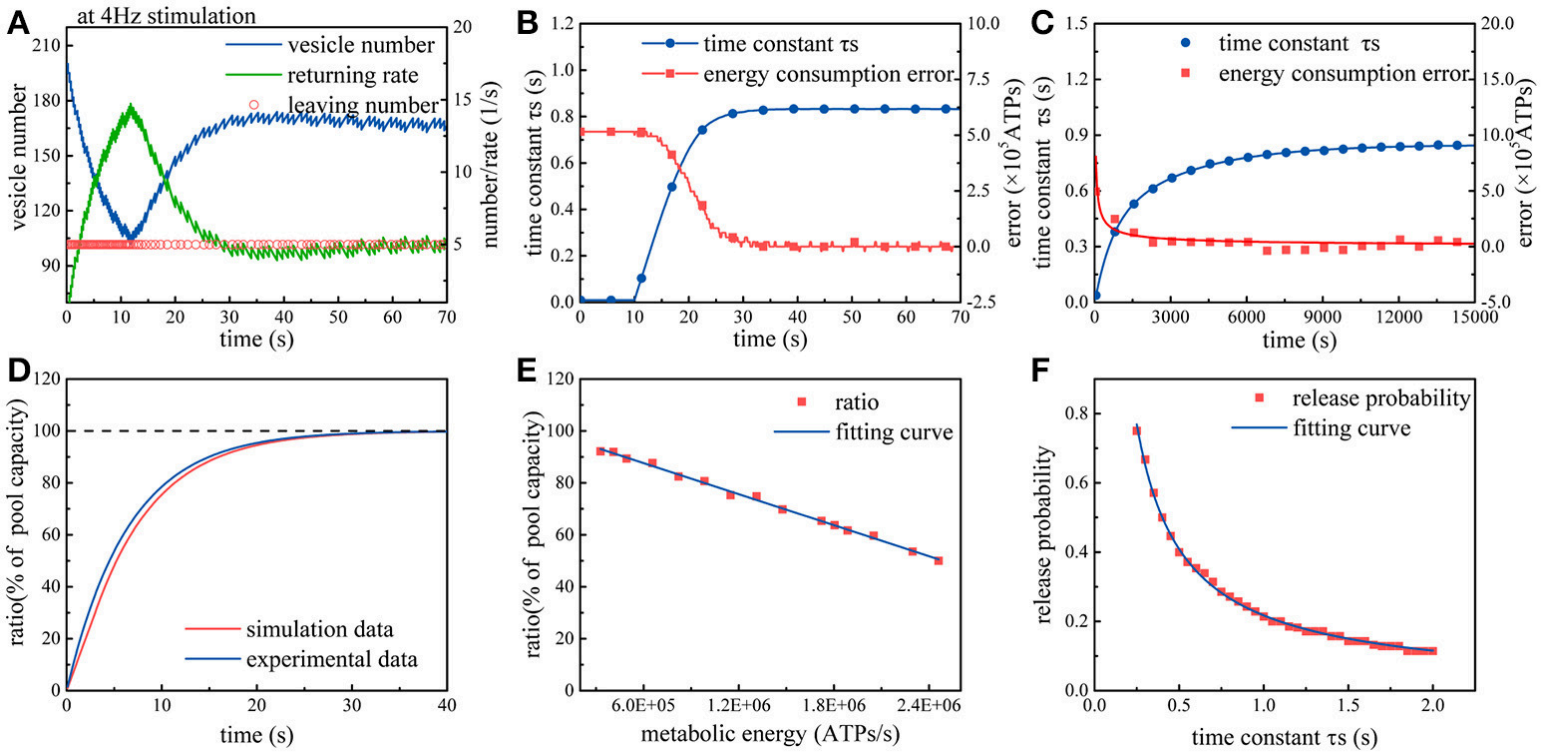
Simulation of the synaptic transmission model with the constraint. **(A)** Due to the constraint, the rate of synaptic vesicles leaving the recycling pool gradually declines to maintain the release probability within the range of 0.25~0.5. The rate of vesicles returning to the recycling pool varies with the rate of vesicles leaving the recycling pool. Once *v*_*s*_ equals *v*_*r*_, the number of synaptic vesicles maintains a dynamic balance. **(B)** Under the constraint, the time constant τ_*s*_ gradually increases so that the difference between the actual metabolic energy consumption and the desired energy consumption (8.2 × 10^5^ ATP/s) tends to zero. **(C)** The model is repeatedly stimulated by action potential sequences. Each stimulation lasts for 50 s. The frequency of action potential sequences in each stimulation obeys an N(4, 1) Gaussian distribution. After the input of approximately three hundred stimuli, the synapse model gradually adapts to the stimulus signal. **(D)** The recovery of the recycling pool in which the vesicles are completely exhausted. The red curve represents the simulation results, and the dark blue curve represents the experimental results (Heidelberger et al., 2002). **(E)** Spike signals with the frequency of 4 Hz are continuously input into the model. When the number of vesicles in the recycling pool reaches dynamic balance, the ratio of the actual vesicle number to the maximum capacity of the pool varies with different synaptic energy level. **(F)** In the case that the frequency of action potential remains unchanged, the larger the time constant τ_*s*_, the smaller the release probability.

Subsequently, we repeat above simulations in the case of considering the effects of synaptic energy. In this case, the model can be optimized according to synaptic energy levels. From Figure 3A, we can find that due to the regulation from synaptic energy, the rate of synaptic vesicles leaving the recycling pool gradually decreases and then remains stable. Clearly, in the first 10 s of the simulation, more than 100 vesicles leave the recycling pool, and then the rate of vesicles leaving the recycling pool gradually decreases to approximately 50 vesicles per 10 s. On the contrary, the return rate of vesicles to the recycling pool increases from zero and then changes with the leaving rate until it is equal to the leaving rate. As we know, the number of vesicles in the recycling pool depends on the leaving rate and return rate. During the initial period of time, the leaving rate is significantly greater than the return rate, so the number of vesicles in the recycling pool drops rapidly. Subsequently, as the return rate gradually rises and equals the leaving rate, the number of vesicles in the recycling pool ceases to drop and maintains dynamic balance. It can be found from Figure 3B that the regulation can continuously narrow the gap between the actual energy consumption and the desired energy consumption at synapses. Besides, during this process, the time constant τ_*s*_ gradually increases and then remains stable. As we know, the larger the time constant τ_*s*_, the more time the vesicles spend on the preparation for exocytosis. Therefore, we can conclude that release probability of synapses adapts to synaptic energy level by regulating the speed of synaptic vesicle cycle. Finally, we repeatedly input action potential sequences whose frequency obeys a Gaussian distribution into the model. It can be found from Figure 3C that under the regulation from synaptic energy, the time constant gradually increases and the difference between the actual energy consumption and desired energy consumption gradually approaches zero. Obviously, the regulation from metabolic energy enables synaptic vesicle cycle to quickly adapt to any given ATP concentration at synapses and have a corresponding release probability.

To validate the proposed model, the simulation results are compared with experimental results. We first set the number of vesicles in the recycling pool to zero and then measure the recovery of vesicles in the recycling pool. The experimental results indicate that the recovery of vesicles in the recycling pool can be fitted as an exponential curve with a time constant 6.5 (Heidelberger et al., 2002). Exactly, our simulation results are consistent with the trend of the exponential curve, as shown in Figure 3D. Full recovery of vesicles in the recycling pool requires about 20 s. Next, the synaptic energy level is set to different values while keeping the input signal unchanged. We find that when the number of vesicles in the recycling pool reaches dynamic balance, the ratio of the actual vesicle number to the maximum capacity of the recycling pool varies with different synaptic energy levels (Figure 3E). As biological experiments reveal, without ATP hydrolysis, synaptic vesicles stop cycling and stay in the recycling pool (Heidelberger et al., 2002). In addition, we also find that when the number of vesicles in the recycling pool reaches dynamic balance, the ratio of the actual vesicle number to the maximum capacity of the pool is generally larger than 40%. When the ratio approaches 40%, it remains almost unchanged even if the synaptic energy level in the model increases continuously. This means that the vesicles in the pool are hard to be exhausted under physiological conditions (Fernándezalfonso and Ryan, 2004). In the end, the time constant τ_*s*_ is set to different values and the corresponding release probability is measured (Figure 3F). Obviously, as the time constant τ_*s*_ increases, the release probability decreases. As experiments and theoretical studies reveal, the release probability which can optimize energy efficiency generally stay within a range of 0.25~0.5 (Heidelberger et al., 2002; Harris et al., 2012). According to our simulation results, the time constant τ_*s*_ should stay within the range of 0.5~1.0 s to ensure release probability stay within a range of 0.25~0.5 (Figure 3F). Then, we can roughly calculate that each vesicle takes about 12 s to complete a cycle. Existing experiments show that the maximum time for reuse of a given vesicle is 13~30 s (Neher, 2010). Based on above analyses, the simulation results obtained by our model is reasonable.

### Dendritic energy efficiency varies nonlinearly with the number of simultaneously active synapses

The rat hippocampal CA1 pyramidal neuron receives a large number of excitatory and inhibitory synapses, but not all of them receive signals from presynaptic neurons at the same time. At a given time, some synapses become active for receiving presynaptic signals, while the rest remain silent (Jeff and Subutai, 2016). Why neurons own thousands of synaptic connections and what factors affect the number of simultaneously active synaptic connections remain unclear.

In the case of different numbers of active synapses, many action potential sequences generated by postsynaptic neurons are recorded in our simulations. The simulations are performed on a traditional multi-compartment model which has been successfully used for capturing the complex firing patterns of neurons (Keren et al., 2005; Saraga et al., 2010; Kispersky et al., 2012). This model contains a set of compartments defined by differential equations, and each compartment involves several gating variables, such as sodium conductance and potassium conductance (see Supplementary Material). For simplicity, all synapses in the model are assumed to be excitatory synapses receiving action potentials at 10 Hz, and their interspike intervals obey a Poisson distribution. According to Equations (11) and (12), the information quantity and energy consumption of a spike train can be calculated.

**Figure 4:**
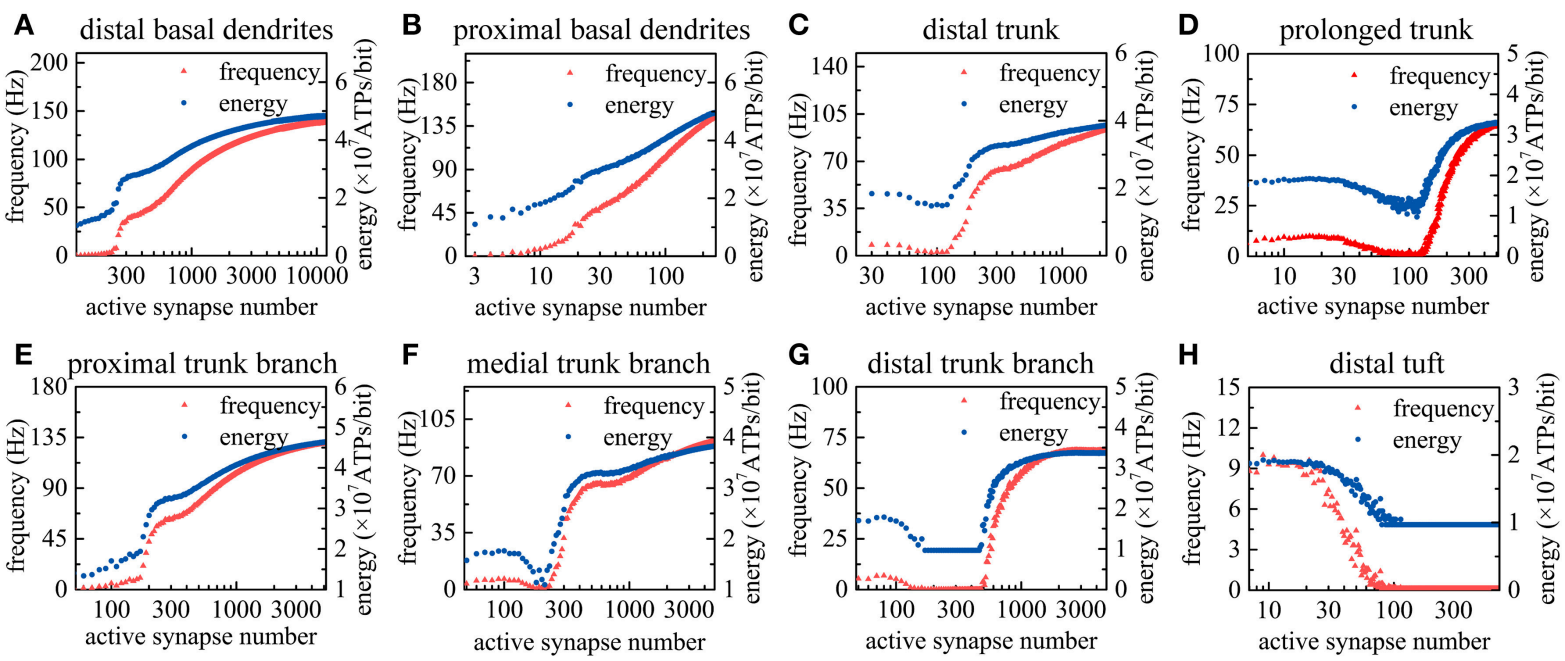
Relations between the action potential frequency and the number of active synapses in individual compartments as well as between the energy consumption of integrating each bit of information and the number of active synapses in individual compartments. Only eight compartments corresponding to the basal dendrites, distal trunk, prolonged trunk, trunk branches, and distal tuft are illustrated here. The values on the horizontal axes indicate the number of active synapses in each compartment. **(A)** The change in the frequency and the energy consumption when the synapses on distal basal dendrites become active. **(B)** The change when the synapses on proximal basal dendrites become active. **(C)** The change when the synapses on distal trunk become active. **(D)** The change when the synapses on prolonged trunk become active. **(E)** The change when the synapses on proximal trunk branch become active. **(F)** The change when the synapses on medial trunk branch become active. **(G)** The change when the synapses on distal trunk branch become active. **(H)** The change when the synapses on distal tuft become active.

As the number of active synapses in a compartment gradually changes, the relations between the number of active synapses and the action potential frequency as well as between the number of active synapses and the energy consumption of integrating each bit of information are shown in Figure 4. Because the majority of excitatory synapses are located in the basal dendrites, distal trunk, prolonged trunk, trunk branches and distal tuft (Table 1), only the compartments corresponding to these dendrites are illustrated in Figure 4. It can be clearly found that as the number of active synapses located in the basal dendrites or the proximal trunk branches increases, the frequency of the action potentials can increase to more than 100 Hz. In contrast, with increasing numbers of active synapses located in other dendrites, such as the distal trunk, prolonged trunk, medial trunk branch, and distal trunk branch, the frequency of action potentials first decreases and then increases but remains less than 100 Hz. As an exception, for increasing numbers of active synapses in the distal tuft, the frequency of action potentials first decreases and then remains constant. Our simulation results have confirmed that (a) a change in the number of active synapses in any compartment can cause a nonlinear change in the frequency of action potentials, and (b) the closer to the AIS the synapses are, the bigger their contribution to the generation of action potentials. Besides, from our simulation results, we can also find that the energy consumption of integrating each bit of information exhibits the same trend as the frequency of action potentials with a change in the number of active synapses in each compartment. That is, the energy consumption of integrating each bit of information is clearly proportional to the frequency of the action potentials.

**Table 1:** Parameters of the pyramidal neuron structural model (Megías et al., 2001).

**Type**	**Percentage of length**	**Diameter (μm)**	**Spine density (/μm)**	**Excitatory input (/μm)**	**Inhibitory input (/μm)**
Proximal basal dendrite	0.033	0.7	0.64	0.64	0.61
Distal basal dendrite	0.33	0.3	3.08	3.08	0.11
Proximal trunk	0.01	2.1	0.03	0.03	1.69
Medial trunk	0.01	2.0	2.37	2.37	0.54
Distal trunk	0.027	1.2	6.98	6.98	0.15
Trunk branches	0.355	0.5	3.52	3.52	0.11
Prolonged trunk	0.025	1.1	1.72	1.72	0.28
Medial tuft	0.053	0.6	0.6	0.69	0.12
Distal tuft	0.157	0.2	0.37	0.48	0.10

**Figure 5:**
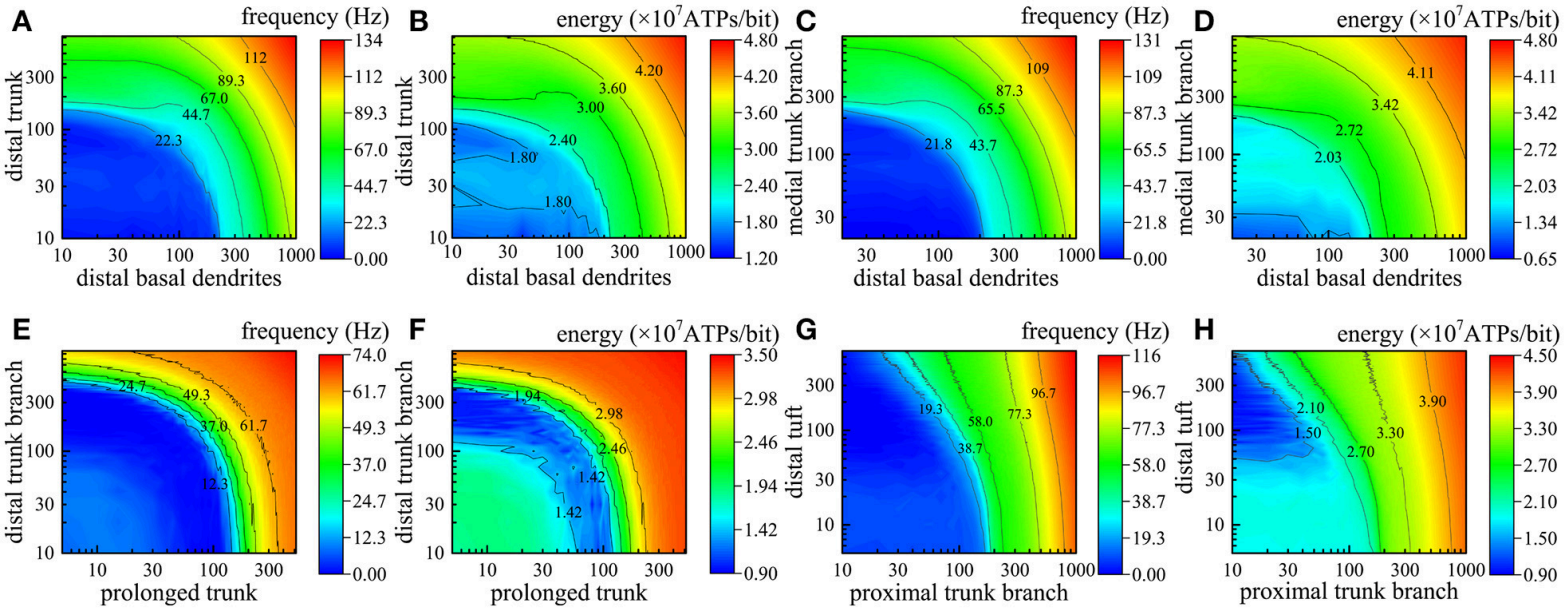
Relations between the action potential frequency and the number of active synapses in two compartments and between the energy consumption of integrating each bit of information and the number of active synapses in two compartments. The horizontal axis and vertical axis indicate the number of active synapses in each compartment. **(A,B)** The change in the frequency and the energy consumption when the synapses on distal basal dendrites and distal truck become active. **(C,D)** The change when the synapses on distal basal dendrites and medial truck branch become active. **(E,F)** The change when the synapses on prolonged trunk and distal truck branch become active. **(G,H)** The change when the synapses on proximal trunk branch and distal tuft become active.

In fact, different numbers of synapses are distributed in different compartments simultaneously. In the next step of our simulations, two or more different compartments are chosen to further study the relations between the action potential frequency and the number of active synapses as well as between the energy consumption of integrating each bit of information and the number of active synapses. The simulation results are illustrated in Figure 5, in which we use different colors to represent the magnitude of action potential frequency and the energy consumption of integrating each bit of information. The closer the color of a region is to red, the higher the frequency of action potentials and the energy consumption of integrating each bit of information; otherwise, the lower the frequency and energy consumption. Obviously, as the number of active synapses located in distal trunk, distal basal dendrites, medial trunk branch, proximal trunk branch, or distal tuft increases, the frequency of action potentials can increase to more than 100 Hz (Figures 5A,C,G). In contrast, with increasing numbers of active synapses located in other dendrites, such as prolonged trunk and distal trunk branch, the frequency of action potentials first decreases and then increases but remains less than 100 Hz (Figure 5E). Besides, we also find that if two compartments are approximately equidistant from the AIS, the equal frequency curves are approximately ¼ of a cycle, as shown in Figure 5E. Otherwise, the equal frequency curves present an irregular shape, as shown in Figures 5A,C,G. In addition, the energy consumption of integrating each bit of information exhibits the same trend as the frequency of action potentials with a change in the number of active synapses (Figures 5B,D,F,H). That is, when the frequency of action potentials is higher, the energy required to integrate one bit of information is greater. It is worth noting that the changes in the frequency and energy consumption caused by the increase in the number of active synapses in different compartments show different trends. The increase in the number of active synapses in some compartments causes an increase in the frequency and energy consumption (e.g., Figures 5B,D), while for other compartments, the frequency and energy consumption first decreases and then increases (Figures 5E,F). The reason why this situation occurs is that action potentials generated in AIS can also propagate back into dendrites (Nevian et al., 2007). In this situation, the current transferred from dendrites to AIS is offset by the current transferred from AIS to dendrites, which results in a decrease in the frequency and energy consumption. This phenomenon is related to many factors, such as the spiking frequency and the feature of compartments, and does not occur in every compartment.

Biological experiments have revealed that most of neurons in CNS generate action potentials at a low frequency (Attwell and Laughlin, 2001; Harris et al., 2012). According to our simulation results, it can be found that the energy consumption of integrating each bit of information is less than 5.0 × 10^7^ ATP. When the action potential frequency is less than 50 Hz, the energy consumption is relatively low, and when the frequency is greater than 50 Hz, the energy consumption increases rapidly. The energy consumption of integrating each bit of information during dendritic integration is proportional to action potential frequency. Therefore, we can naturally infer that the limited metabolic energy may not allow individual neurons to process information with high-frequency action potentials, and only a part of the synapses receive presynaptic signals during a given period so that neurons have a low action potential frequency. Although each pyramidal neuron receives thousands of excitatory synapses in the dendritic tree, only a few synapses receive presynaptic signals at the same time. These findings raise the question of why neurons have these morphological and integrative properties. We learn from the above analysis that although a large number of active synaptic connections can effectively generate action potentials, a large amount of metabolic energy is consumed. Therefore, a trade-off is needed between the number of active connections and the consumption of metabolic energy (Niven et al., 2007).

## Discussion and conclusions

### Metabolic energy could exert regulation on synaptic vesicle cycle of neurons

In this paper, based on the latest research findings, we propose a biologically plausible synaptic transmission model that enables us to vividly simulate the process of action potentials inducing synaptic vesicles to release neurotransmitters. In addition, from the perspective of optimization principles, we derive an essential equation which can describe the relationship between synaptic vesicle cycle and synaptic energy level. Based on this essential equation, the synaptic vesicle cycle in our model changes synaptic energy level, and synaptic energy level in turn regulates the synaptic vesicle cycle, which is one of the contributions in our work.

The synaptic vesicle cycle is simulated without and with the effects of synaptic energy, respectively. There are huge differences between the simulation results in both cases. In the case of not considering the effects of metabolic energy, the release probability of synapses exhibits large fluctuations as the frequency of input signals changes. Undoubtedly, this is inconsistent with biological experimental findings that the release probability of most synapses stay within a range of 0.25~0.5 to optimize synaptic energy efficiency (Harris et al., 2012). Conversely, when considering the effects of metabolic energy in the model, the cycle of synaptic vesicles becomes stable. Regardless of how the frequency of input signals changes, release probability could always stay within the range of 0.25~0.5. Therefore, it is clear that the metabolic energy at synapses not only drives synaptic vesicle cycle, but also participates in the regulation of this cycle. From the simulation results, it also can be found that the time constant τ_*s*_ continually changes as the release probability gradually approaches to the range of 0.25~0.5. As we know, the time constant τ_*s*_ determines how long vesicles spend on the preparation for exocytosis. Therefore, we can further conclude that release probability of synapses adapts to energy level by regulating the speed of synaptic vesicle cycle. In addition, compared with previous studies, another contribution in our work is that the proposed model can simulate synaptic vesicle cycle under different conditions and even show how synaptic energy regulate the cycle of synaptic vesicles. We study the recovery of depleted recycling pools, as well as the cycle of synaptic vesicles under different ATP concentrations. The simulation results are in good agreement with the biological experiment results, which means that our model is biologically plausible.

However, these findings raise some questions about what biological mechanisms probably account for the adaptation of synaptic vesicle cycle to synaptic energy levels. The adaptability of synaptic vesicle cycle is probably caused by a variety of biochemical reactions, such as the production of soluble adhesion protein complexes, the movement of motor proteins, and the metabolic cooperation between astrocytes and neurons, etc. During the process of synaptic vesicles moving from the recycling pool to the RRP and preparing for exocytosis, with the participation of calcium ions, proteins on vesicle membrane bind to proteins on active zones to produce soluble adhesion protein complexes, which need ATP hydrolysis to provide energy (Heidelberger et al., 2002; Südhof, 2013). In addition, the transportation of synaptic vesicles and protein requires the participation of motor proteins, powered by the hydrolysis of ATPs (Heidelberger et al., 2002). Undoubtedly, biochemical reactions that heavily depend on the hydrolysis of ATPs are also affected by ATP concentration. Increasing evidence suggests that astrocytes are the major source of the energetic substrates used by the synapses and play important roles in the regulation of synaptic transmission (Newman, 2003; Jourdain et al., 2007; Perea and Araque, 2007; Magistretti and Allaman, 2015; Bazargani and Attwell, 2016). It has also been demonstrated that metabolic energy plays an important role in neuronal synaptic functions through constraints (Göbel et al., 2010; Rangaraju et al., 2014). As a consequence, from the perspective of engineering, all neural mechanisms mentioned above lead us to infer that synaptic metabolic energy exerts constraints on synaptic vesicle cycle of neurons. Assuming that synapses initially have a high release probability, high-frequency action potentials could induce a large number of synaptic vesicles to release neurotransmitters in a short time, leading to a sharp decrease in metabolic energy at the synapses. Because the ability of astrocytes to supply energetic substrates to neuronal terminals is limited, a metabolic energy shortage could occur if a large number of synaptic vesicles were to release neurotransmitters within a short time. In this situation, the production of soluble adhesion protein complexes as well as the movement of motor proteins will decelerate, and synaptic vesicles will require a longer time to prepare for exocytosis. For some arriving action potentials, then, no synaptic vesicles will be available. Accordingly, the release probability at the synapses will decrease. Conversely, for a synapse with a very low release probability, sufficient energy can promote the activities of the synaptic vesicles and increase the release probability.

Recently, Lu et al. point out that the synapses with high release probability show a more energy- efficient design for releasing neurotransmitters (Lu et al., 2016). This seems to be contrary to the viewpoint that most of synapses maintain a low release probability to obtain optimal energy efficiency. However, it is worth noting that the energy efficiency in Lu's study is computed only according to presynaptic energy consumption, while postsynaptic energy consumption is neglected. Experimental and theoretical studies have shown that postsynaptic energy consumption far exceeds presynaptic energy consumption during the release of neurotransmitters (Attwell and Laughlin, 2001; Howarth et al., 2012). In our model, both presynaptic and postsynaptic energy consumption are taken into account when computing the energy efficiency. Obviously, the energy efficiency in Lu's study actually refers to presynaptic energy efficiency, whereas the energy efficiency in our model refers to the energy efficiency of a synapse.

Existing studies have shown that a low release probability can optimize the energy efficiency of the synapse (Levy and Baxter, 2002; Harris et al., 2012). Lu's study also suggests that if presynaptic terminals really consume a negligible proportion of synaptic energy budget, low release probability synapses undoubtedly gain a greater advantage (Lu et al., 2016). According to Lu's study, a high release probability is needed for presynaptic terminals to obtain optimal energy efficiency. However, as our model reveals, when the action potential frequency remains unchanged, a higher release probability can lead to a greater postsynaptic energy consumption, and the magnitude of postsynaptic energy consumption is greater than that of presynaptic energy consumption. When high release probability makes presynaptic terminals reach the optimal energy efficiency, the energy efficiency of the synapse will decrease due to the greater postsynaptic energy consumption. Once the energy efficiency of the synapse decreases, synaptic vesicle cycle is immediately regulated and the release probability decreases accordingly. This may be a reason why the release probability corresponding to the optimal energy efficiency of the synapse is lower than the release probability corresponding to presynaptic optimal energy efficiency.

### Metabolic energy is one of the determinants of the number of simultaneously active synapses

In addition to synaptic transmission, dendritic integration is also an important but metabolically expensive step in information processing in pyramidal neurons. Therefore, based on the simulation of a multi-compartment model in pyramidal neurons, we also quantitatively study the relationship between dendritic integration and metabolic energy. In the case of different numbers of active synapses at different compartments, we record the corresponding frequency of action potentials and the energy consumption of integrating each bit of information. From our simulation results, we show that (a) a change in the number of active synapses in any compartment can cause a nonlinear change in the frequency of action potentials, and (b) the closer to the AIS the synapses are, the bigger their contribution to the generation of action potentials. Our simulation results also confirm that the energy consumption of integrating each bit of information is clearly proportional to the frequency of the action potentials.

Our simulation results show that when the action potential frequency is less than 50 Hz, the energy consumption is relatively low, and when the frequency is greater than 50 Hz, the energy consumption increases rapidly. Exactly, many studies have revealed that most of neurons in CNS fire at a low frequency (Attwell and Laughlin, 2001; Harris et al., 2012). Therefore, from the perspective of energy, the limited metabolic energy may not allow individual neurons to process information using high-frequency action potentials. To match the neural energy level, only a part of the synapses receive presynaptic signals during a given period so that the neurons have a low action potential frequency. That is, an excessive number of active synapses over a period of time could increase the energy burden on pyramidal neurons. This finding raise a question why neurons have large number of synapses but only a part of the synapses receive presynaptic signals over a period of time. Studies have shown that the number of connection patterns in pyramidal neurons increases with the number of synapses, and a large number of connection patterns provides powerful information processing capability for neurons (Jeff and Subutai, 2016). Therefore, combing with our simulation results, we can infer that the number of synapses is a trade-off between the metabolic energy and the connection patterns of pyramidal neurons. There is also another question about what biological mechanisms probably account for the adaptation of the number of synapses to neural energy levels. The answers may be found through a further understanding for the roles of astrocytes. Astrocytes emerge as essential participants in nearly all aspects of neural development, such as promoting synapse formation and pruning exuberant synaptic connections (Corty and Freeman, 2013). Under the guidance of a unified principle, the nervous system may manage synaptic connections through astrocytes.

In the proposed models, we regard the energy consumption of synaptic transmission and dendritic integration as a constant, which is reasonable for the neurons at low firing frequency. However, synaptic transmission and dendritic integration are not instantaneous, and the corresponding energy would not be consumed immediately. Therefore, there should be an applicable frequency range of the proposed model. For dendritic integration, a single action potential can last 1~2 ms, during which metabolic energy is consumed to reset membrane potential (Dayan and Abbott, 2001). This means that the energy consumption corresponding to an action potential also lasts 1~2 ms. Besides, given that the refractory period of action potentials also lasts a few milliseconds (Dayan and Abbott, 2001), it can be roughly estimated that the applicable frequency range for the multi-compartment model is 0~100 Hz. For synaptic transmission, the majority of energy is consumed by postsynaptic action of the released neurotransmitters (Attwell and Laughlin, 2001). Neurotransmitters lead to the activation of the receptor channels on the postsynaptic membrane, and a large number of ions pour into the postsynaptic membrane through these channels. To pump out these ions, much energy is consumed. During releasing neurotransmitters of a single vesicle, the average open time of non-NMDA channels is ~1.5 ms, while the average open time of NMDA channels is ~50 ms (Attwell and Laughlin, 2001). Therefore, it can be roughly estimated that the applicable frequency range for the synaptic transmission model is 0~20 Hz.

## Author contributions

YY and TF conceived the project and designed the experiments. All authors contributed to the development of the concepts presented in the paper. YY performed the experiments. YY and TF performed the data analysis. All authors helped write the manuscript.

### Conflict of interest statement

The authors declare that the research was conducted in the absence of any commercial or financial relationships that could be construed as a potential conflict of interest.
