# Supplementary material for: Effects of Metabolic Energy on Synaptic Transmission and Dendritic Integration in Pyramidal Neurons
Source: Front Comput Neurosci. 2018 Sep 26;12:79. doi: 10.3389/fncom.2018.00079 (PMC6168642; doi:10.3389/fncom.2018.00079)
Supplement: Supplementary file 1 [file Table_1.docx]

# Effects of metabolic energy on synaptic transmission and dendritic integration in pyramidal neurons

**Ye Yuan^1,2^, Hong Huo^1,2*^ & Tao Fang^1,2*^**

^1^Department of Automation, Shanghai Jiao Tong University, Shanghai 200240, China

^2^Key Laboratory of System Control and Information Processing, Ministry of Education, Shanghai 200240, China

*** Correspondence:**

Tao Fang [tfang@sjtu.edu.cn](mailto:tfang@sjtu.edu.cn)

Hong Huo [huohong@sjtu.edu.cn](mailto:huohong@sjtu.edu.cn)

## Supplementary materials

**Multi-compartment model.** The dendritic morphology of hippocampal CA1 pyramidal neurons varies across species and cortical regions, but almost all such neurons have numerous short basal dendrites and one long apical dendrite bifurcating into oblique dendritic branches([Megías et al., 2001](#_ENREF_11); [Altemus et al., 2005](#_ENREF_2)). Due to differences between morphological structures, such as the distance from the cell body, the spine density, and the shaft diameter, dendritic integration shows significant location dependence([Magee, 2000](#_ENREF_9); [Williams and Stuart, 2003](#_ENREF_17)). That is, the same signals received by synapses can have different effects at the AIS. To examine the relations between the number of synaptic inputs and metabolic energy during dendritic integration and between the frequency of action potentials and metabolic energy during dendritic integration, we construct a multi-compartment model based on hippocampal CA1 pyramidal neurons, in which dendrites with similar morphological structures are represented by a single compartment (Fig. 6). Thirteen compartment models in a single pyramidal neuron are established, including the distal tuft, proximal tuft, prolonged trunk, distal trunk, medial trunk, proximal trunk, trunk branches, proximal basal dendrites, distal basal dendrites, cell body, and axonal initial segment. The trunk branches are represented by three compartments, namely, the branches linked to the distal trunk, medial trunk, and proximal trunk. The structural parameters of these compartments are listed in Table 1.

The membrane of pyramidal neurons contains large quantities of channel proteins, which are the structural basis of transmembrane ion movement. The kinetics of different types of ion channels vary widely. Ion flux depends heavily on factors such as the kinetics and distribution of ion channels and the dendritic morphology([Reyes, 2001](#_ENREF_13); [Major et al., 2013](#_ENREF_10)). Here, only seven major types of ion channels are considered, as follows([Reyes, 2001](#_ENREF_13); [Keren et al., 2005](#_ENREF_6)): a hyperpolarization-activated cation current channel (H), a Na^+^ channel (Na), a fast inactivating K^+^ channel (Kf), a slow inactivating K^+^ channel (Ks), a small-conductance Ca^2+^-gated K^+^ channel (Ksk), a large-conductance Ca^2+^-gated K^+^ channel (Kbk), and a Ca^2+^ channel (Ca). The channel activation and inactivation kinetics are described in Table 2.


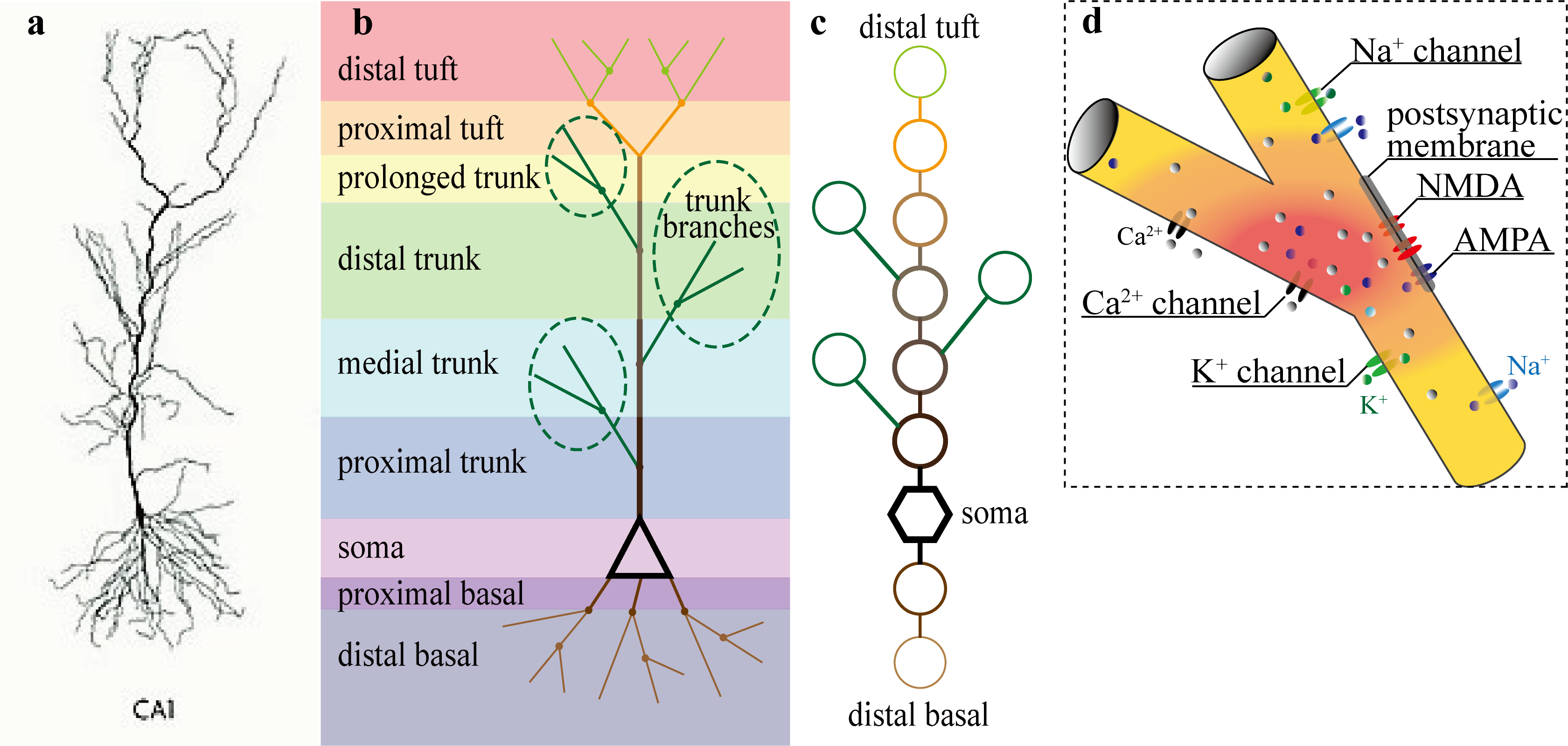


**Figure 1 | Structural schematic of pyramidal neurons.** **a**, The morphological structures of pyramidal neurons in the hippocampal CA1 cortical areas([Spruston, 2008](#_ENREF_16)). **b**, The dendrites of pyramidal neurons are divided into nine parts: the distal tuft, proximal tuft, prolonged trunk, distal trunk, medial trunk, proximal trunk, trunk branches, proximal basal dendrites, and distal basal dendrites, which are depicted in unique colours. The trunk branches are enclosed in dashed ellipses, and the other structures are placed in eight different coloured bands. Detailed data on the dendritic branches are given in Table 1. **c**, Each compartment is represented as a circle of the same colour used in a. **d**, Dendrites actually resemble hollow pipelines, and many channel proteins and receptors are distributed on the wall, such as Na+ channels, K+ channels, NMDA receptors, and AMPA receptors. Higher ion concentrations are represented by deeper colours of the neuron membrane. The membrane potential of the orange-red region is higher than that of the yellow region.

The mathematical descriptions of each compartment model are modified from the traditional Hodgkin–Huxley model ([Dayan and Abbott, 2001](#_ENREF_3)) as follows

$$C_{m}\frac{dV_{i}}{dt}=-I_{ions}^{i}-I_{nei}^{i}-I_{sys}^{i}$$

where *V_i_* is the membrane potential of the i-th compartment in the model. The ion current *I_ions_*, coupling current *I_nei_*, and input currents *I_sys_* from synapses all contribute to the rise in the membrane potential and trigger action potentials. The ion current is generated by various ions passing through the channels mentioned above. The coupling current is the sum of the currents between the i-th compartment and its adjacent compartments, which are computed as follows

$$I_{nei}^{i}=\sum_{j=1}^{N_{i}} g_{c}\cdot\left(V_{i}-V_{j}\right)$$

$$\begin{matrix} I_{ions}^{i}=g_{h}\cdot m\cdot\left(V_{i}-E_{h}\right)+g_{Na}\cdot m^{3}\cdot h\cdot\left(V_{i}-E_{Na}\right) \\ \text{ }+g_{Kf}\cdot r^{2}\cdot\left(s_{1}+s_{2}\right)/2\cdot\left(V_{i}-E_{Kf}\right)+g_{Ks}\cdot a^{4}\cdot b\cdot\left(V_{i}-E_{Ks}\right) \\ \text{ }+g_{Ksk}\cdot m\cdot\left(V_{i}-E_{Ksk}\right)+g_{Kbk}\cdot\alpha^{3}\cdot\gamma^{2}\cdot\beta\cdot\left(V_{i}-E_{Kbk}\right) \\ \text{ }+g_{Ca}\cdot\alpha^{2}\cdot\beta\cdot\left(V_{i}-E_{Ca}\right) \end{matrix}$$

$$I_{sys}^{i}=g_{ex}\cdot\left(V_{i}-E_{ex}\right)+g_{inh}\cdot\left(V_{i}-E_{inh}\right)$$

$$\tau_{ex}\cdot\frac{dg_{ex}}{dt}=-g_{ex},\text{ }\tau_{inh}\cdot\frac{dg_{inh}}{dt}=-g_{inh}$$

where N*_i_* is the number of compartments adjoining the i-th compartment. The coupling conductance([Pinsky and Rinzel, 1995](#_ENREF_12); [Yi et al., 2014](#_ENREF_18)) is *g_c_* = 10 pS/μm^2^. The conductance of the ion channels is as follows (in pS/μm^2^): dendrites([Schaefer et al., 2003](#_ENREF_15); [Keren et al., 2005](#_ENREF_6); [Kispersky et al., 2012](#_ENREF_7)): *g_Na_* = 81, *g_Kf_* = 28, *g_Ks_* = 6.13, *g_Ksk_* = 0.65, *g_Kbk_* = 1.85, and *g_Ca_* = 1.5; soma: *g_Na_* = 284, *g_Kf_* = 294, *g_Ks_* = 220, *g_Ksk_* = 3.05, *g_Kbk_* = 1.93, and *g_Ca_* = 3. The kinetic equations of kinetic variables such as *m*, *n*, and *h*, are defined in the Table 2. The equilibrium potentials (in mV) are *E_H_* = -30, *E_Na_* = 60, *E_K_* = -80, *E_Kf_* = -80, *E_Ks_* = -80, *E_Ksk_* = -80, *E_Kbk_* = -80, and *E_Ca_* = 130. Notably, the distribution of hyperpolarization-activated cation current channels *g_h_* is not uniform and increases exponentially with distance([Kole et al., 2006](#_ENREF_8)). Depending on the distance from the soma, the *g_h_* of compartments varies from 2.5 to 100 pS/μm^2^. The membrane capacitance *C_m_* is set to 0.009 pF/μm^2^. The values *g_ex_* and *g_inh_* are the conductance of the excitatory and inhibitory synapses, and their reversal potentials (in mV) are *E_ex_* = 0 and *E_inh_* = -80, respectively([Guillamon et al., 2006](#_ENREF_4)). When an action potential arrives at the synapses, *g_ex_* and *g_inh_* increase by *Δg_ex_* and *Δg_inh_*, respectively. Otherwise, both parameters obey Equation (17), in which $\tau_{ex}$ and $\tau_{inh}$ are set to 5 and 10, respectively. Here, *Δg_ex_* and *Δg_inh_* are set to 3500 and 1500 pS/synapse, respectively([Rudolph et al., 2004](#_ENREF_14)). The multi-compartment model is implemented in C/C++, in which the Runge-Kutta numerical method is used. The code is available upon request.

Table 1 Channel activation and inactivation kinetics([Keren et al., 2005](#_ENREF_6); [Keren et al., 2009](#_ENREF_5); [Almog and Korngreen, 2014](#_ENREF_1))

| Channel type | Equation |
| --- | --- |
| $H$ | $\begin{matrix} g_{h}=g_{h,max}\cdot m \\ \tau_{m}\cdot\frac{dm}{dt}=-m+m_{\infty} \\ m_{\infty}=1/\left(1+e^{\left(V+91\right)/6}\right),\tau_{m}=1/\left(0.0004e^{-0.025V}\text{+}0.088e^{0.062V}\right) \end{matrix}$ |
| $Na$ | $\begin{matrix} g_{Na}=g_{Na,\max}\cdot m^{3}\cdot h \\ \tau_{m}\cdot\frac{dm}{dt}=-m+m_{\infty}\text{，}\tau_{h}\cdot\frac{dh}{dt}=-h+h_{\infty} \\ \text{ }m_{\infty}=1/\left[1+e^{-\left(V+38\right)/10}\right],\tau_{m}=0.058\text{+0.114}e^{-\left[\left(V+36\right)/28\right]^{2}} \\ h_{\infty}=1/\left[1+e^{\left(V+66\right)/6}\right],\tau_{h}=0.28\text{+16.7}e^{-\left[\left(V+60\right)/25\right]^{2}} \end{matrix}$ |
| $K_{s}$ | $\begin{matrix} g_{Ks}=g_{Ks,\max}\cdot r^{2}\cdot\left(s_{1}+s_{2}\right)/2 \\ \tau_{r}\cdot\frac{dr}{dt}=-r+r_{\infty},\text{ }\tau_{r}=1/\alpha_{r}+\beta_{r},\text{ }r_{\infty}=\alpha_{r}/\left(\alpha_{r}+\beta_{r}\right) \\ \alpha_{r}=0.0052\cdot\left(V_{m}-11.1\right)/\left[1-e^{-\left(V_{m}-11.1\right)/13.1}\right]\text{，}\beta_{r}=0.02\cdot e^{-\left(V_{m}+1.27\right)/71}-0.005\text{，} \\ \tau_{s1}\cdot\frac{ds_{1}}{dt}=-s_{1}+s_{1,\infty},\text{ }\tau_{s2}\cdot\frac{ds_{2}}{dt}=-s_{2}+s_{2,\infty}, \\ \tau_{s1}=360+\left[1010+23.7\cdot\left(V_{m}+54\right)\right]\cdot e^{-\left[\left(V_{m}+75\right)/48\right]^{2}},\text{ }\tau_{s2}=2350+1380\cdot e^{-0.011\cdot V_{m}}-210\cdot e^{-0.03\cdot V_{m}} \\ s_{1,\infty}=s_{2,\infty}=1/\left[1+e^{\left(V_{m}+58\right)/11}\right] \end{matrix}$ |
| $K_{f}$ | $\begin{matrix} g_{Kf}=g_{Kf,\max}\cdot a^{4}\cdot b \\ \tau_{a}\cdot\frac{da}{dt}=-a+a_{\infty},\text{ }\tau_{b}\cdot\frac{db}{dt}=-b+b_{\infty}, \\ a_{\infty}=1/\left[1+e^{-\left(V_{m}+47\right)/29}\right],\tau_{a}=0.34+0.92\cdot e^{-\left[\left(V_{m}+71\right)/59\right]^{2}} \\ b_{\infty}=1/\left[1+e^{\left(V_{m}+66\right)/10}\right],\tau_{b}=8+49\cdot e^{-\left[\left(V_{m}+73\right)/23\right]^{2}} \end{matrix}$ |
| $K_{SK}$ | $\begin{matrix} g_{KSK}=g_{Ksk,\max}\cdot m \\ \tau_{m}\cdot\frac{dm}{dt}=-m+m_{\infty} \\ \tau_{m}=1/\left(1.3^{4}\cdot\left[Ca^{2+}\right]_{i}^{4}\right),m_{\infty}=\left(1.3^{4}\cdot\left[Ca^{2+}\right]_{i}^{4}\right)/\left(1.3^{4}\cdot\left[Ca^{2+}\right]_{i}^{4}+0.06\right) \end{matrix}$ |
| $K_{BK}$ | $\begin{matrix} g_{KBK}=g_{KBK,\max}\cdot\alpha^{3}\cdot\gamma^{2}\cdot\beta\\ \tau_{\alpha}\cdot\frac{d\alpha}{dt}=-\alpha+\alpha_{\infty},\text{ }\tau_{\beta}\cdot\frac{d\beta}{dt}=-\beta+\beta_{\infty},\text{ }\tau_{\gamma}\cdot\frac{d\gamma}{dt}=-\gamma+\gamma_{\infty}, \\ \alpha_{\infty}=1/\left[1+e^{-\left(V_{m}+29\right)/6.2}\right],\tau_{\alpha}=1000.505/e^{\left(V_{m}+86\right)/10} \\ \gamma_{\infty}=1/\left(1+0.001/\left[Ca^{2+}\right]_{i}\right),\tau_{\gamma}=1,\beta_{\infty}=0.085+0.915/\left[1+e^{\left(V_{m}+32\right)/6}\right],\tau_{\beta}=1001.9/e^{-\left(V_{m}+49\right)/13} \end{matrix}$ |
| $Ca$ | $\begin{matrix} g_{Ca}=g_{Ca,\max}\cdot\alpha^{2}\cdot\beta\\ \tau_{\alpha}\cdot\frac{d\alpha}{dt}=-\alpha+\alpha_{\infty},\text{ }\tau_{\beta}\cdot\frac{d\beta}{dt}=-\beta+\beta_{\infty} \\ \alpha_{\infty}=1/\left[1+e^{-\left(V_{m}+55\right)/7.4}\right],\tau_{\alpha}=3+1/\left[e^{(V_{m}+60)/20}+e^{-\left(V_{m}+135\right)/15}\right] \\ \beta_{\infty}=1/\left[1+e^{-\left(V_{m}+75\right)/5}\right],\tau_{\beta}=30+1/\left[e^{(V_{m}+60)/4}+e^{-\left(V_{m}+425\right)/50}\right] \end{matrix}$ |

## References

Almog, M., and Korngreen, A. (2014). A quantitative description of dendritic conductances and its application to dendritic excitation in layer 5 pyramidal neurons. *Journal of Neuroscience the Official Journal of the Society for Neuroscience* 34(1)**,** 182-196.

Altemus, K.L., Lavenex, P., Ishizuka, N., and Amaral, D.G. (2005). Morphological characteristics and electrophysiological properties of CA1 pyramidal neurons in macaque monkeys. *Neuroscience* 136(3)**,** 741-756.

Dayan, P., and Abbott, L.F. (2001). Theoretical Neuroscience. *Mit Press*.

Guillamon, A., Mclaughlin, D., and Rinzel, J. (2006). Estimation of synaptic conductances. *Journal of Physiology-Paris* 100(1–3)**,** 31-42.

Keren, N., Baryehuda, D., and Korngreen, A. (2009). Experimentally guided modelling of dendritic excitability in rat neocortical pyramidal neurones. *Journal of Physiology* 587(Pt 7)**,** 1413.

Keren, N., Peled, N., and Korngreen, A. (2005). Constraining compartmental models using multiple voltage recordings and genetic algorithms. *Journal of Neurophysiology* 94(6)**,** 3730.

Kispersky, T.J., Caplan, J., and Marder, E. (2012). Increase in Sodium Conductance Decreases Firing Rate and Gain in Model Neurons. *Journal of Neuroscience the Official Journal of the Society for Neuroscience* 32(32)**,** 10995-11004.

Kole, M.H.P., Hallermann, S., and Stuart, G.J. (2006). Single Ih Channels in Pyramidal Neuron Dendrites: Properties, Distribution, and Impact on Action Potential Output. *Journal of Neuroscience the Official Journal of the Society for Neuroscience* 26(6)**,** 1677-1687.

Magee, J.C. (2000). Dendritic integration of excitatory synaptic input. *Nature Reviews Neuroscience* 1(3)**,** 181-190.

Major, G., Larkum, M.E., and Schiller, J. (2013). Active Properties of Neocortical Pyramidal Neuron Dendrites. *Annual Review of Neuroscience* 36(1)**,** 1.

Megías, M., Emri, Z., Freund, T.F., and Gulyás, A.I. (2001). Total number and distribution of inhibitory and excitatory synapses on hippocampal CA1 pyramidal cells. *Neuroscience* 102(3)**,** 527-540.

Pinsky, P.F., and Rinzel, J. (1995). Intrinsic and network rhythmogenesis in a reduced Traub model for CA3 neurons. *Journal of Computational Neuroscience* 2(3)**,** 275-275.

Reyes, A. (2001). INFLUENCE OF DENDRITIC CONDUCTANCES ON THE INPUT-OUTPUT PROPERTIES OF NEURONS. *Annual Review of Neuroscience* 24(1)**,** 653-675.

Rudolph, M., Piwkowska, Z., Badoual, M., Bal, T., and Destexhe, A. (2004). A method to estimate synaptic conductances from membrane potential fluctuations. *Journal of Neurophysiology* 91(6)**,** 2884-2896.

Schaefer, A.T., Larkum, M.E., Sakmann, B., and Roth, A. (2003). Coincidence detection in pyramidal neurons is tuned by their dendritic branching pattern. *Journal of Neurophysiology* 89(6)**,** 3143-3154.

Spruston, N. (2008). Pyramidal neurons: dendritic structure and synaptic integration. *Nature Reviews Neuroscience* 9(3)**,** 206-221.

Williams, S.R., and Stuart, G.J. (2003). Role of dendritic synapse location in the control of action potential output. *Trends in Neurosciences* 26(3)**,** 147.

Yi, G.S., Wang, J., Wei, X.L., Tsang, K.M., Chan, W.L., Deng, B., et al. (2014). Exploring how extracellular electric field modulates neuron activity through dynamical analysis of a two-compartment neuron model. *Journal of Computational Neuroscience* 36(3)**,** 383.
